# Dr. Trotter, in Reply to Dr. Yeats

**Published:** 1800-06

**Authors:** T. Trotter

**Affiliations:** Plymouth Dock


### *To the Editors of the Medical and Phyſical Journal.*

GENTLEMEN,

THE laſt remarks of Dr. Yeats, in Number XV. of your Journal, are juſt come to hand.—The Doctor's language in one inſtance appears exceptionable: What does he mean by a "manly recantation?" In my ſecond Volume, I have admitted, that there were miſtakes in my letter to Mr. Nepean; and in p. 39, there is ſuch an apology made as the error ſeemed to require; for it does not affect the ground which I had taken in this diſpute. I might have, with equal propriety, retained the term *nitrous gas*, as *nitrous vapour.* Is not Dr. Yeats contending that this vapour oxygenates the atmoſphere, or bodies floating in it? If it gives out oxygen, what is the reſiduum? In what ſtate of oxydation is the *azote* left? Is it *nitrous gas*, or *azotic gas?* If either of theſe, a poiſonous quality, according to his own premiſes, muſt ſtill be added to the ſurrounding medium.*

But I deny that this vapour increaſes the reſpirable portion, on the very authorities which Dr. Yeats adduces. That *oxygen* may be ſeparated, no one can doubt; but this ſeparation muſt

---

* Does not this exactly agree with our firſt remark, that it adds to the atmoſphere of a ſhip's deck the very ſubſtance which every intelligent officer is conſtantly endeavouring to expel?

muſt be ſpontaneous, if it is to add to the vital part of the air, and make it more ſalutary to life: it muſt form combinations with no other ſubſtance, but remain in a condition fit to be inſpired by the human lungs. Therefore, neither Berthollet's experiments, nor Lavoiſier's opinion, ſanction the concluſion that the vapour of *nitric acid* adds to the reſpirable portion of the atmoſphere; and by no means apply to the proceſs of diffuſing it in the decks of ſhips, and the wards of hoſpitals. Whatever may have been Dr. Yeats's firſt opinion on this ſpecies of fumigation, it is uncontroverted that the original author intended to deſtroy *contagious matter*. It therefore involves the whole hiſtory of fumigation; which, to trace *ab origine*, includes the ludicrous inſtances of necromancy, and does not degrade the gravity of diſcuſſion. We there follow the human mind, in its progreſs through ages of barbariſm and ſuperſtition, to the attempt of ingrafting a popular deluſion on the ſound ſtock of human knowledge, that has been accumulated by chaſte and accurate experiment.

If ſuch is the defence of this once prevailing and unqueſtioned doctrine, in the hands of Dr. Yeats, what are we to think of the *farrago* for fumigation, ſtill employed by many phyſicians of the preſent day? Surely, it is a more natural practice to look to the common atmoſphere in its pure ſtate, for the healthful ſupply of *oxygen*, than to ſeek it from chemical agents. How many pounds of nitre will give out *oxygen* equal to the quantity admitted by a port or window, or the draught of a well trimmed wind-ſail? I think with Dr. Yeats, that "fumigations, as formerly employed, are more calculated to deceive by enveloping diſagreeable ſmells, than to be beneficial by neutralizing and deſtroying contagious miaſma." But this does not agree with what he ſays afterwards: "If we can by the powerful aid of chemical agents neutralize diſagreeable exhalations, is it not a deſirable object? No: it was much better to ſhift the poacher, and burn the ſtraw on which he lay in Bedford Jail, than to neutralize the offenſive exhalations by *nitrous vapour*. In the laſt ſentence quoted, Dr. Yeats ſhows much of that diſpoſition which we have obſerved in all the favourers of fumigation; he recurs to it inſenſibly. He wants *oxygen* to deſtroy a diſagreeable ſmell; and ſeems to forget, that by opening the window he can have it in abundance: *quem quæris adeſt*: he does not appear to like it in this form, but goes round by the Cape of Good Hope, and brings it from the ſoil of Hindoſtan in an Eaſt Indiamen. He thus leaves the preventive of a court phyſician to the indelicate office of purifying foul utenſils, which a cleanly nurſe at Haſlar Hoſpital, or a decent London chamber-maid, would effectually correct by

ſoap

ſoap and water, and throwing up the ſaſhes; and that too, long before a fumigating phyſician would be able to arrange his pipkins, to the entire diſcredit of this new-fangled prophylactic, and the utter confuſion of all medical necromancy.

Dr. Yeats will allow with me, that all proceſſes of this ſort carry with them an air of myſtery, and are calculated to impoſe on by-ſtanders, and thus render attendants of the ſick negligent and careleſs of the moſt eſſential parts of their office. Theſe vapours alſo convert the white-waſh of our decks into a *nitrate of lime*, that makes the ſurface of the timber attract moiſture in damp weather, which we abhor.

I muſt now conclude, that it is an object of national importance to convince the officer and ſeaman, by the moſt ſimple manner poſſible, that they have in their own hands the genuine prevention and cure of infection. This has, in fact, been the caſe; and I have the notion that a large portion of human miſery has been oppoſed or relieved by it; perhaps, more ſucceſsfully during the laſt three weeks of our duty than on any former occaſion, in clearing two ſhips of the line of contagion and fever. The detail of this buſineſs muſt be reſerved for future animadverſion; becauſe, what I conſider as highly gratifying to every medical reader, it will unfold the *triumph* of ſcientific arrangements, that are capable of extending ſympathy, truly Britiſh, to the ſick bed of a brave ſailor, over a vitious ſyſtem of tactics.

In thus taking leave of Dr. Yeats, I beg to aſſure him of my perfect eſteem, and the high opinion I entertain of his learning and talents. I muſt alſo, Gentlemen, beg your pardon, and that of your Readers, for the haſty ſcrawls I have ſent you. I am, with great reſpect,

GENTLEMEN,

Your very obedient and humble ſervant,

T. TROTTER.

*Plymouth Dock,*

